# Birth Order Differences in First-Year Neurodevelopment

**DOI:** 10.1001/jamanetworkopen.2026.1265

**Published:** 2026-03-06

**Authors:** Akiko Tsuchida, Kenta Matsumura, Haruka Kasamatsu, Tomomi Tanaka, Kei Hamazaki, Hidekuni Inadera

**Affiliations:** 1 Department of Public Health, Faculty of Medicine, University of Toyama, Toyama, Japan; 2 Toyama Regional Center for JECS, University of Toyama, Toyama, Japan; 3 Graduate School of Health Sciences, Aomori University of Health and Welfare, Aomori, Japan; 4 Department of Pediatrics, Faculty of Medicine, University of Toyama, Toyama, Japan; 5 Department of Public Health, Gunma University Graduate School of Medicine, Maebashi, Japan

## Abstract

This cohort study examines whether firstborn and second-born children differ in neurodevelopmental outcomes during infancy.

## Introduction

Birth order differences in cognitive and developmental outcomes are well documented, with firstborn children typically outperforming their later-born siblings.^1^ Several explanations have been proposed, including parental learning and changes in family circumstances. In infancy, when parental engagement plays a central role, resource dilution has been suggested as a contributing mechanism.^2,3^ However, only a few studies have compared siblings within the same family, particularly in the first year of life, to separate birth-order factors from stable family factors. We, therefore, examined whether firstborn and second-born children differ in neurodevelopmental outcomes during infancy using a mother fixed-effects design.

## Methods

This cohort study used a within-family design to compare pairs of firstborn and second-born singleton children enrolled in the Japan Environment and Children’s Study (JECS).^4^ Ethical approval was obtained from relevant institutional review boards, and written informed consent was collected. This study was conducted in accordance with STROBE reporting guidelines.

Participants who became pregnant twice within the JECS recruitment period could re-enroll, enabling identification of sibling pairs. The key birth characteristics of JECS participants, such as sex ratio, preterm birth rate, and low birth weight rate, closely resemble those of the Japanese Vital Statistics.^5^

Neurodevelopment was evaluated using the Ages & Stages Questionnaires, Third Edition (ASQ-3) at 6 and 12 months. Participants were compensated with gift certificates (valued at ¥1000 for each questionnaire and ¥3000 for biological sample donation). Parental engagement was measured using a 5-item composite score (range, 0-18, with higher scores indicating greater engagement) capturing the frequency of parent-child play, outdoor activities, and reading aloud (eAppendix and eFigure in Supplement 1). Birth order differences were estimated using mother fixed-effects linear models, comparing siblings within the same family and removing all shared, time-invariant family characteristics. Models included child sex and birth year indicators. SEs were clustered at the mother level. Parental engagement was analyzed using analogous fixed-effects models. Given the conceptual limitations of causal mediation within fixed-effects designs, we did not conduct formal mediation analysis; instead, parental engagement differences are presented descriptively within the same framework. Analyses were conducted using SAS statistical software version 9.4 (SAS Institute) and R statistical software version 4.5.1 (R Project for Statistical Computing). Statistical significance was defined as 2-sided *P* < .05. Estimates were derived from fixed-effects linear models, and 95% CIs were used to assess statistical significance.

## Results

A total of 2117 sibling dyads were included. Estimated marginal means indicated that second-born children scored lower than firstborns across multiple ASQ-3 domains at both 6 and 12 months (Table 1). At 6 months, differences ranged from 1.9 points in communication to 13.8 points in personal-social development. At 12 months, differences persisted for fine motor and personal-social domains, whereas estimates for communication and problem-solving were small and not statistically significant. Sensitivity analyses adjusting for birth weight yielded nearly identical results (changes ≤0.1 points).

**Table 1.** Estimates From a Mother Fixed-Effects Model of Ages & Stages Questionnaires, Third Edition, Domain Scores by Birth Order Among 2117 Sibling Dyads

Domain	Age 6 mo		Age 12 mo	
	EMM (SE)^a^	Mean difference, β (95% CI)^b^	EMM (SE)^a^	Mean difference, β (95% CI)^b^
Communication				
Firstborns	47.7 (0.6)	0 [Reference]	38.4 (0.8)	0 [Reference]
Second-borns	45.8 (0.5)	−1.9 (−3.7 to −0.1)^c^	36.5 (0.8)	−1.9 (−4.3 to 0.6)
Gross motor				
Firstborns	35.9 (0.8)	0 [Reference]	44.7 (1.2)	0 [Reference]
Second-borns	29.5 (0.7)	−6.4 (−8.9 to −4.0)^c^	41.2 (1.1)	−3.6 (−7.1 to 0.02)
Fine motor				
Firstborns	43.2 (0.9)	0 [Reference]	49.8 (0.9)	0 [Reference]
Second-borns	37.2 (0.9)	−5.9 (−8.8 to −3.1)^c^	45.9 (0.8)	−3.9 (−6.8 to −1.1)^c^
Problem-solving				
Firstborns	46.7 (0.9)	0 [Reference]	41.5 (0.9)	0 [Reference]
Second-borns	40.1 (0.8)	−6.7 (−9.3 to −4.0)^c^	43.3 (0.8)	1.7 (−1.1 to 4.6)
Personal-social				
Firstborns	39.4 (0.8)	0 [Reference]	39.5 (0.9)	0 [Reference]
Second-borns	25.6 (0.9)	−13.8 (−16.7 to −10.9)^c^	34.8 (0.9)	−4.8 (−7.7 to −1.9)^c^

Abbreviation: EMM, estimated marginal mean.

^a^ SEs are clustered at the mother level.

^b^ Mother fixed-effects models were adjusted for child sex and birth year indicators, accounting for within-mother clustering and all shared, time-invariant family factors.

^c^ Indicates a primary contrast.

Parental engagement scores were also lower for second-born children (Table 2). Fixed-effects models indicated a mean difference of −0.77 points (95% CI, −1.26 to −0.29 points) after adjustment for sex and birth year indicators.

**Table 2.** Mother Fixed-Effects Estimates of Birth Order Differences in Parental Engagement

Variable^a^	Mean difference, β (95% CI)^b^
Birth order, second-born vs firstborn	−0.77 (−1.25 to −0.29)^c^
Sex, female vs male	0.01 (−0.13 to 0.16)
Birth year	
2012 vs 2011	−0.30 (−0.54 to −0.06)^c^
2013 vs 2011	−0.30 (−0.76 to 0.16)
2014 vs 2011	−0.25 (−0.83 to 0.34)

^a^ Reference categories are firstborn birth order, male sex, and birth year 2011.

^b^ Estimates are from mother fixed-effects models with SEs clustered at the mother level.

^c^ Indicates a primary contrast.

## Discussion

Birth order differences favoring firstborn children are well established,^1,2,3^ and the findings of this cohort study extend this evidence by demonstrating that such differences appear during the first year of life. Parallel reductions in parental engagement for second-born infants are consistent with the resource dilution model, suggesting that reduced individualized parental time may contribute to early developmental variation. Some differences attenuated by 12 months, which may be partly explained by the confluence model^6^: later-born infants may benefit from observing and interacting with older siblings, compensating for reduced parental resources.

Limitations include that ASQ-3 scores are parent reported and may reflect birth order–related differences in parental perception. Although all ASQ-3 domains are scored on the same scale, differences varied across domains at 6 months, with the personal-social domain showing the largest gap. These differences narrowed by 12 months, and their long-term clinical meaning remains uncertain. In conclusion, this within-family analysis shows that small but consistent birth order differences in neurodevelopment are already observable during the first year of life.

## Notes

Supplement 1. eAppendix. Supplemental Methods eFigure. Distribution of Parental Engagement Scores eReferences

Supplement 2. Nonauthor Collaborators. Japan Environment and Children’s Study Group

Supplement 3. Data Sharing Statement

## References

[1] Belmont L, Marolla FA. Birth order, family size, and intelligence. Science. 1973;182(4117):1096-1101. doi:10.1126/science.182.4117.1096 4750607

[2] Blake J. Family size and the quality of children. Demography. 1981;18(4):421-442. doi:10.2307/2060941 7308532

[3] Price J. Parent-child quality time: does birth order matter? J Hum Resour. 2008;43(1):240-265. doi:10.3368/jhr.43.1.240

[4] Kawamoto T, Nitta H, Murata K, ; Working Group of the Epidemiological Research for Children’s Environmental Health. Rationale and study design of the Japan Environment and Children’s Study (JECS). BMC Public Health. 2014;14:25. doi:10.1186/1471-2458-14-25 24410977 PMC3893509

[5] Michikawa T, Nitta H, Nakayama SF, ; Japan Environment and Children’s Study Group. Baseline profile of participants in the Japan Environment and Children’s Study (JECS). J Epidemiol. 2018;28(2):99-104. doi:10.2188/jea.JE20170018 29093304 PMC5792233

[6] Zajonc RB, Sulloway FJ. The confluence model: birth order as a within-family or between-family dynamic? Pers Soc Psychol Bull. 2007;33(9):1187-1194. doi:10.1177/0146167207303017 17586733

