# Supplementary material for: Birth Order Differences in First-Year Neurodevelopment
Source: JAMA Netw Open. 2026 Mar 6;9(3):e261265. doi: 10.1001/jamanetworkopen.2026.1265 (PMC12966919; doi:10.1001/jamanetworkopen.2026.1265)
Supplement: Supplement 1. — eAppendix. Supplemental Methods eFigure. Distribution of Parental Engagement Scores eReferences [file jamanetwopen-e261265-s001.pdf]

Supplemental Online Content

Tsuchida A, Matsumura K, Kasamatsu H, Tanaka T, Hamazaki K, Inadera H; Japan Environment and Children's Study Group. Birth order differences in first-year neurodevelopment. *JAMA Netw Open*. 2026;9(3):e261265. doi:10.1001/jamanetworkopen.2026.1265

eAppendix. Supplemental Methods
eFigure. Distribution of Parental Engagement Scores
eReferences

This supplemental material has been provided by the authors to give readers additional information about their work.

© 2026 Tsuchida A et al. *JAMA Network Open*.

eAppendix. Supplemental Methods

Study Design

The Japan Environment and Children's Study (JECS) is a birth cohort study that investigates the associations between environmental factors and childhood health. Participant recruitment for the study took place at 15 regional centers in Japan from 2011 to 2014 and involved a face-to-face explanation of the study to pregnant women. Written informed consent was obtained from the enrolled participants. The JECS study design has been reported previously.[1-3]

Study Data

The present study analyzed data from the National Institute for Environmental Studies dataset provision website, which was initially published in January 2025 and downloaded in March 2025. Most participants enrolled once and provided their information, as well as that of their children, along with the timeline. However, if participants gave birth and became pregnant within the recruitment period, they could re-register for the younger child to participate in the same manner as the older child. To obtain the participants for the within-family comparison of those with multiple participations and those with singleton births, from the data of 98 233 singleton live births, we excluded 58 128 records due to missing information on birth order or because they involved the third or later birth of a child. We also excluded 465 records with a discrepancy between birth order and social rank, 33 224 with only a single enrollment, and 2182 with missing Ages & Stages Questionnaires®, Third Edition (ASQ-3) data at 6 months or 1 year of age. Thus, data from 2117 dyads (ie, a total of 4234 registrants) of the first 2 non-twin offspring from the same mothers were analyzed. Missing data on ASQ-3 scores at 6 months or 1 year of age resulted in the exclusion of 2182 records prior to analysis.

Measurements

To collect data on demographics and socioeconomic status, all mothers completed a self-administered questionnaire on 5 occasions: in the first (M-T1) and second/third (M-T2) trimesters and at 1 (M-1m), 6 (C-6m), and 12 (C-1y) months postpartum. In addition, we obtained data on past birth history from medical record transcripts. The birth order of the children who participated in this study was determined by referring to the previous births in the medical record transcripts (Dr-T1). The birth order and social rank in the family may have differed for several reasons, including the presence of adopted children or stepchildren or the death of older siblings. In this study, social rank was determined using information on "children living together" in the questionnaire completed by the mothers in the first trimester (M-T1). Only participants whose birth order and social rank were in perfect agreement were included in this study.
Neurodevelopment was evaluated using the ASQ-3, a parent-completed child-monitoring questionnaire that is a validated scale recommended by UNICEF for assessing neurological development in children.[4,5] The ASQ-3 comprises 21 age-specific, structured questions divided among the following 5 domains: communication, gross motor skills, fine motor skills, problem-solving, and personal–social skills. Each participating parent, generally the mother, was administered the ASQ-3 when her child was 6 months and 1 year old.
Data on participants' demographics and socioeconomic status were obtained by self-administered questionnaires during early and mid-late pregnancy. The following information on parental engagement during infancy was collected from a questionnaire that asked about the condition of the child at 6 months of age (C-6m) or 1 year of age (C-1y). Parent engagement was assessed using a composite score ranging from 0 to 18, based on 5 parent-reported items related to involvement with the child. These items included playing with the child, going out together, and reading picture books and were reported separately for mothers and fathers. Higher scores indicated greater parental involvement in daily interactions with the child.

© 2026 Tsuchida A et al. *JAMA Network Open*.

Construction of the Parent Engagement Score

The parent engagement score was constructed by summing the responses to 5 items derived from caregiver questionnaires administered at 6 months and 1 year of age. Two items were obtained from the 6-month questionnaire, which assessed the partner's (typically the father's) involvement in childcare over the past month:

1. Frequency of playing with the child indoors
2. Frequency of taking the child outside to play

The responses for both items were rated on a 4-point Likert scale (0 = never, 1 = seldom, 2 = sometimes, 3 = all the time).

Three additional items were obtained from the 1-year questionnaire, based on the primary caregiver's (typically the mother's) report:

3. Frequency of playing with the child
4. Frequency of going out with the child, excluding childcare drop-offs and pick-ups
5. Frequency of reading picture books aloud to the child

The responses for these items were scored on a 5-point scale (0 = seldom, 1 = 1–3 times/month, 2 = 1–2 times/week, 3 = 3–4 times/week, 4 = ≥ 5 times/week).

The distribution of the parent engagement score was examined among participants without missing engagement data in the full sample prior to eligibility screening (n = 85,371). The distribution was right-skewed, with a mean of 13.5 (SD = 2.5). The score ranged from 0 to 18, with the 25th, 50th, and 75th percentiles at 12, 14, and 15, respectively.

Statistical Analysis

Neurodevelopmental outcomes were assessed in each ASQ-3 developmental domain (communication, gross motor, fine motor, problem-solving, and personal–social) at 6 months and 12 months of age. Birth-order differences were estimated using mother fixed-effects linear models, which compare siblings within the same family and remove all shared, time-invariant family factors. This approach restricts inference to within-family differences while accounting for stable characteristics such as socioeconomic background, parenting style, and genetics shared by siblings.

Each model included indicators for child sex and birth year to adjust for secular or cohort-related differences between siblings. Standard errors were clustered at the mother level to adjust for non-independence of observations within sibling pairs. Estimated marginal means for first- and secondborn children were obtained from these models to facilitate interpretation.

Parental engagement was analyzed analogously using mother fixed-effects linear models with the same adjustment set (sex and birth year). Given the conceptual limitations of causal mediation analyses in fixed-effects frameworks—particularly the violation of the sequential ignorability assumption and the risk of bias resulting from adjustment for mediators that may act as intermediates rather than confounders—we did not conduct formal mediation analysis. Instead, birth-order differences in parental engagement are reported descriptively within the same modeling framework.

All statistical analyses were conducted using SAS version 9.4 (SAS Institute Inc.) and R (R Foundation for Statistical Computing).

© 2026 Tsuchida A et al. *JAMA Network Open*.

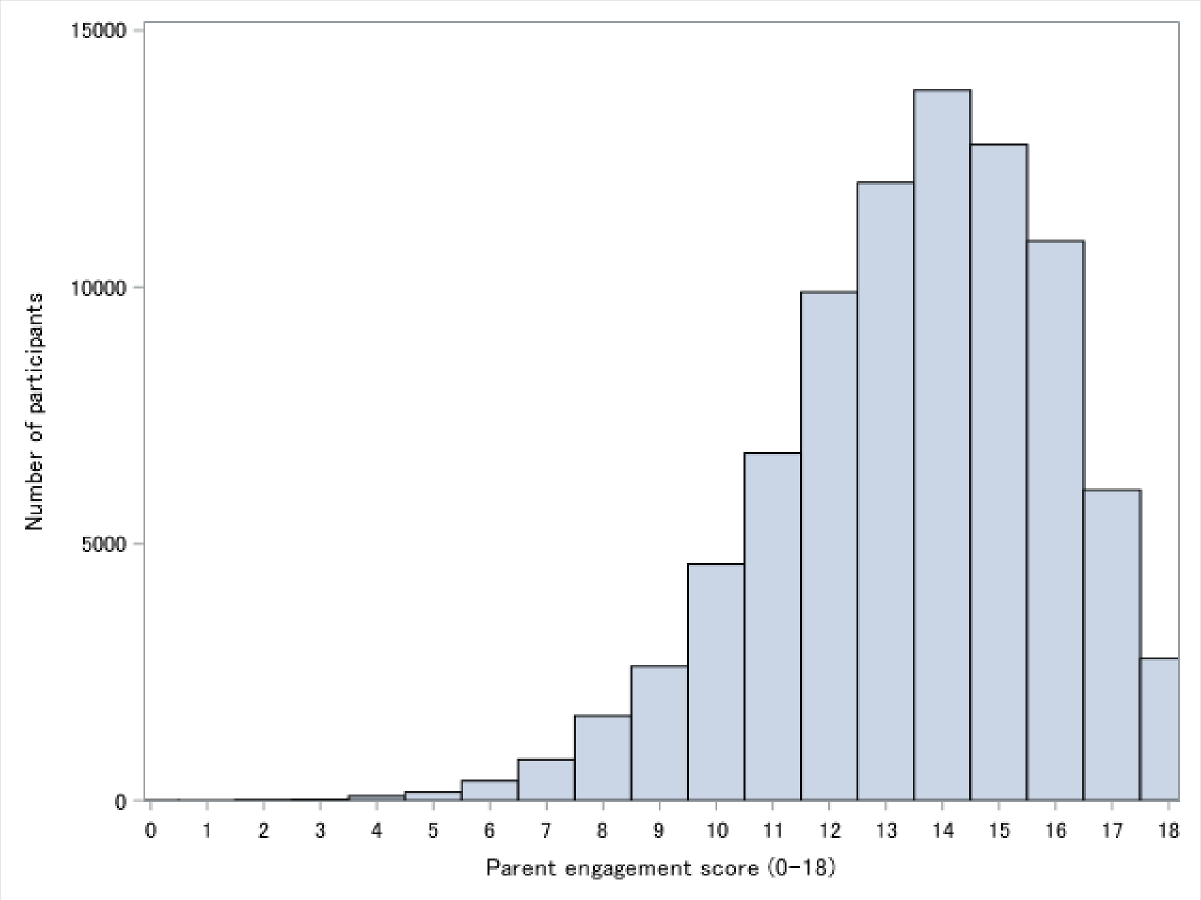

eFigure. Distribution of Parental Engagement Scores
The histogram shows the distribution of the parental engagement scores among participants without missing data in the full sample prior to eligibility screening (n = 85,371).

eReferences

1. Kawamoto T, Nitta H, Murata K, et al. Rationale and study design of the Japan environment and children's study (JECS). *BMC Public Health.* 2014;14:25.
2. Michikawa T, Nitta H, Nakayama SF, et al. Baseline Profile of Participants in the Japan Environment and Children's Study (JECS). *J Epidemiol.* 2018;28(2):99-104.
3. Iwai-Shimada M, Nakayama SF, Isobe T, et al. Questionnaire results on exposure characteristics of pregnant women participating in the Japan Environment and Children Study (JECS). *Environ Health Prev Med.* 2018;23(1):45.
4. Romero Otalvaro AM, Granana N, Gaeto N, et al. ASQ-3: Validation of the Ages and Stages Questionnaire for the detection of neurodevelopmental disorders in Argentine children. *Arch Argent Pediatr.* 2018;116(1):7-13.
5. Mezawa H, Aoki S, Nakayama SF, et al. Psychometric profile of the Ages and Stages Questionnaires, Japanese translation. *Pediatr Int.* 2019;61(11):1086-1095.

© 2026 Tsuchida A et al. *JAMA Network Open*.
