# Supplementary material for: Birth Order Differences in First-Year Neurodevelopment
Source: JAMA Netw Open. 2026 Mar 6;9(3):e261265. doi: 10.1001/jamanetworkopen.2026.1265 (PMC12966919; doi:10.1001/jamanetworkopen.2026.1265)
Supplement: Supplement 2. — Nonauthor Collaborators. Japan Environment and Children’s Study Group [file jamanetwopen-e261265-s002.pdf]

Supplemental Online Content: Nonauthor Collaborators
*First name, last name, and suffix (if applicable) are required and will appear in PubMed.

*Group Name: Japan Environment and Children's Study Group

***First Name and Middle Initial(s)**	***Last Name**	***Suffix (eg, Jr, III)**	Academic Degrees	Institution	Location (city, state/province, country)	Role or Contribution, eg, chair, principal investigator	Group (if more than 1 Group listed in the byline) and/or Subgroup (eg, Steering Committee)
Shin	Yamazaki			National Institute for Environmental Studies	Tsukuba, Ibaraki, Japan		
Yukihiro	Ohya			National Center for Child Health and Development	Tokyo, Japan		
Reiko	Kishi			Hokkaido University	Sapporo, Hokkaido, Japan		
Nobuo	Yaegashi			Tohoku University	Sendai, Miyagi, Japan		
Koichi	Hashimoto			Fukushima Medical University	Fukushima, Fukushima, Japan		
Chisato	Mori			Chiba University	Chiba, Chiba, Japan		
Shuichi	Ito			Yokohama City University	Yokohama, Kanagawa, Japan		
Zentaro	Yamagata			University of Yamanashi	Chuo, Yamanashi, Japan		
Michihiro	Kamijima			Nagoya City University	Nagoya, Aichi, Japan	Principal investigator	
Takeo	Nakayama			Kyoto University	Kyoto, Kyoto, Japan		
Hiroyasu	Iso			Osaka University	Suita, Osaka, Japan		
Masayuki	Shima			Hyogo College of Medicine	Nishinomiya, Hyogo, Japan		
Youichi	Kurozawa			Tottori University	Yonago, Tottori, Japan		
Narufumi	Suganuma			Kochi University	Nankoku, Kochi, Japan		
Koichi	Kusuhara			University of Occupational and Environmental Health	Kitakyushu, Fukuoka, Japan		
Takahiko	Katoh			Kumamoto University	Kumamoto, Kumamoto, Japan		
