# Supplementary material for: Birth Order Differences in First-Year Neurodevelopment
Source: JAMA Netw Open. 2026 Mar 6;9(3):e261265. doi: 10.1001/jamanetworkopen.2026.1265 (PMC12966919; doi:10.1001/jamanetworkopen.2026.1265)
Supplement: Supplement 3. — Data Sharing Statement [file jamanetwopen-e261265-s003.pdf]

Data Sharing Statement

Tsuchida. Birth Order Differences in First-Year Neurodevelopment. *JAMA Netw Open*. Published March 06, 2026. doi:10.1001/jamanetworkopen.2026.1265

Data
Data available: No

Additional Information
Explanation for why data not available: Explanation for why data not available: Data are unsuitable for public deposition due to ethical restrictions and the legal framework of Japan. It is prohibited by the Act on the Protection of Personal Information (Act No. 57 of May 30, 2003, amendment on September 09, 2015) to publicly deposit data containing personal information. Ethical Guidelines for Medical and Health Research Involving Human Subjects enforced by the Japan Ministry of Education, Culture, Sports, Science and Technology and the Ministry of Health, Labour and Welfare also restricts the open sharing of epidemiologic data. All inquiries about access to data should be sent to: jecs-en@nies.go.jp. The person responsible for handling inquiries sent to this e-mail address is Dr. Shoji F. Nakayama, JECS Programme Office, National Institute for Environmental Studies.
